# Supplementary material for: Suicide before and during the COVID-19 Pandemic: A Systematic Review with Meta-Analysis
Source: Int J Environ Res Public Health. 2023 Feb 14;20(4):3346. doi: 10.3390/ijerph20043346 (PMC9960664; doi:10.3390/ijerph20043346)
Supplement: Supplementary file 1 [file ijerph-20-03346-s001.zip › ijerph-2157738-supplementary.pdf]

Supplementary Material

Figure S1. Sensitivity analysis for non-clinical samples reporting suicidal ideation (leave-one-out method)

Figure S2. Funnel plot for non-clinical samples reporting suicidal ideation

Figure S3. Sensitivity analysis for clinical samples reporting suicidal ideation (leave-one-out method)

Figure S4. Sensitivity analysis for clinical samples reporting suicidal ideation (exclude lower-quality samples)

Figure S5. Funnel plot for clinical samples reporting suicidal ideation

Figure S6. Sensitivity analysis for non-clinical samples reporting suicide attempt (leave-one-out method)

Figure S7. Sensitivity analysis for non-clinical samples reporting suicide attempt (exclude lower-quality samples)

Figure S8. Funnel plot for non-clinical samples reporting suicide attempt

Figure S9. Sensitivity analysis for clinical samples reporting suicide attempt (leave-one-out method)

Figure S10. Sensitivity analysis for clinical samples reporting suicide attempt (exclude lower-quality samples)

Figure S11. Funnel plot for clinical samples reporting suicide attempt

Figure S12. Sensitivity analysis for samples reporting suicide death (leave-one-out method)

Figure S13. Sensitivity analysis for samples reporting suicide death (exclude lower-quality samples)

Figure S14. Funnel plot for samples reporting suicide death

Table S1. PRISMA checklist

Table S2. Risk of bias assessment

Table S3. Summary of meta-regression for non-clinical and clinical samples reporting suicidal ideation

Table S4. Summary of meta-regression for non-clinical and clinical samples reporting suicide attempt

Table S5. Summary of meta-regression for samples reporting death by suicide

File S1. Full search strategy

Figure S1. Sensitivity analysis for non-clinical samples reporting suicidal ideation (leave-one-out method)

Suicidal ideation (non-clinical)

Study name	Point	Lower limit	Upper limit	p-Value
An et al., 2022	1.153	1.017	1.308	0.026
Ayuso-Mateos et al., 2021	1.147	1.020	1.289	0.021
Bountress et al., 2022	1.198	1.069	1.342	0.002
Brailovskaia et al., 2021	1.114	0.992	1.251	0.068
Brausch et al., 2022	1.123	0.999	1.262	0.052
Danielsen et al., 2022	1.162	1.031	1.310	0.014
Ettman et al., 2020	1.077	0.968	1.198	0.171
Gratz et al., 2021	1.161	1.032	1.306	0.013
H. Kim et al., 2022	1.152	1.022	1.299	0.020
Horita et al., 2022a	1.159	1.030	1.304	0.014
Horita et al., 2022b	1.151	1.023	1.295	0.020
Kasal et al., 2022a	1.112	0.990	1.248	0.073
Kasal et al., 2022b	1.102	0.983	1.235	0.095
Kim et al., 2021	1.161	1.027	1.313	0.017
King et al., 2022	1.127	1.002	1.266	0.046
Knudsen et al., 2021a	1.139	1.014	1.280	0.029
Knudsen et al., 2021b	1.145	1.019	1.287	0.023
Knudsen et al., 2021c	1.140	1.015	1.281	0.027
Koenig et al., 2021	1.155	1.028	1.299	0.016
Lee & Hong, 2022a	1.159	1.021	1.315	0.023
Lee & Hong, 2022b	1.152	1.009	1.314	0.036
Liu et al., 2022	1.127	1.003	1.267	0.044
Mayne et al., 2021	1.139	1.012	1.282	0.032
Nichter et al., 2021	1.163	1.034	1.309	0.012
Reuter et al., 2021	1.151	1.023	1.294	0.019
Sivertsen et al., 2022	1.129	1.013	1.259	0.029
Zhang et al., 2020	1.135	1.009	1.277	0.035
Zhu et al., 2021	1.155	1.026	1.301	0.017
	1.142	1.018	1.282	0.024

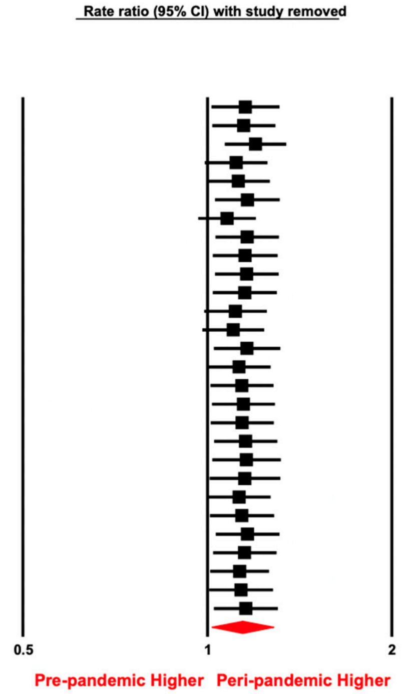

Meta Analysis

Figure S2. Funnel plot for non-clinical samples reporting suicidal ideation

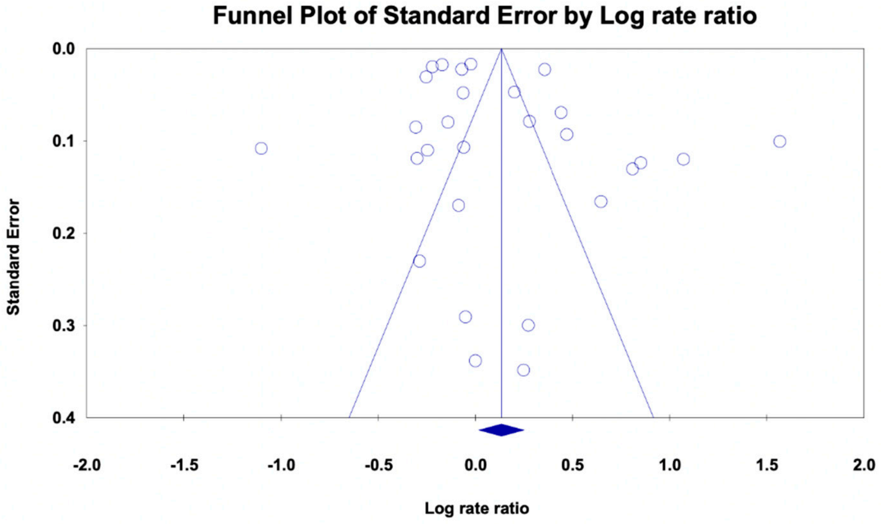

Figure S3. Sensitivity analysis for clinical samples reporting suicidal ideation (leave-one-out method)

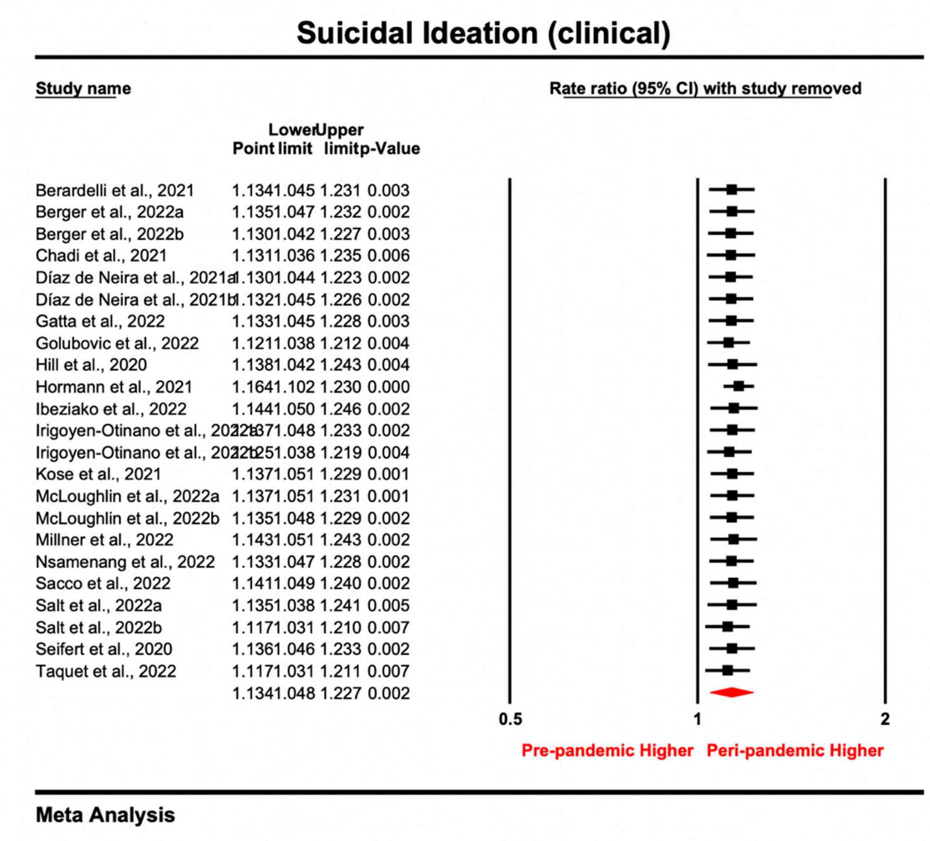

Figure S4. Sensitivity analysis for clinical samples reporting suicidal ideation (exclude lower-quality samples)

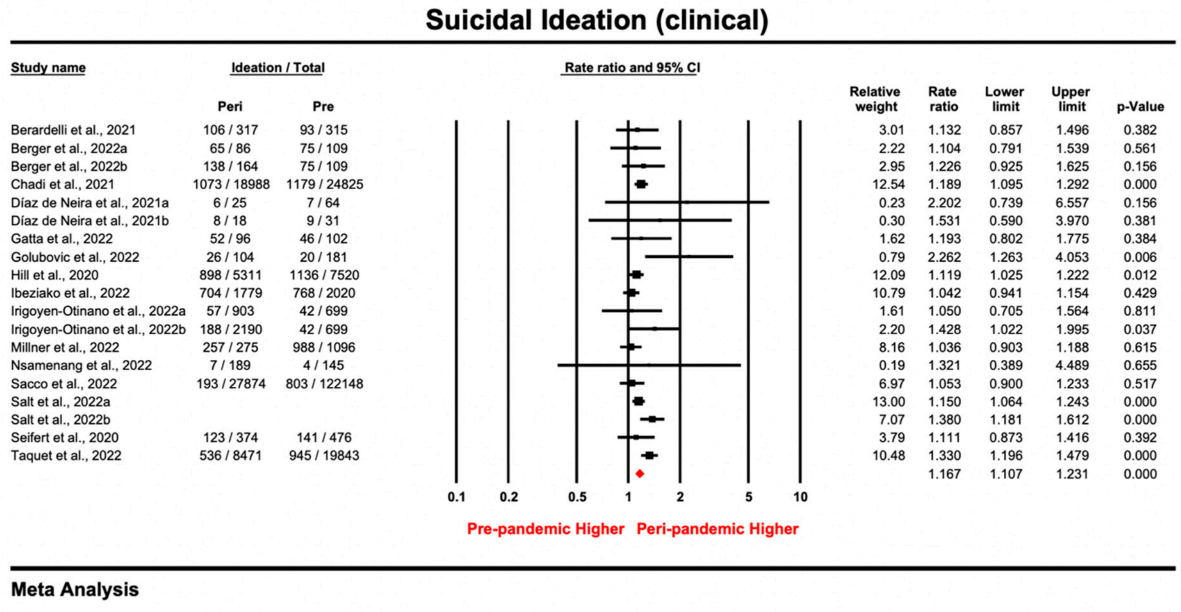

Figure S5. Funnel plot for clinical samples reporting suicidal ideation

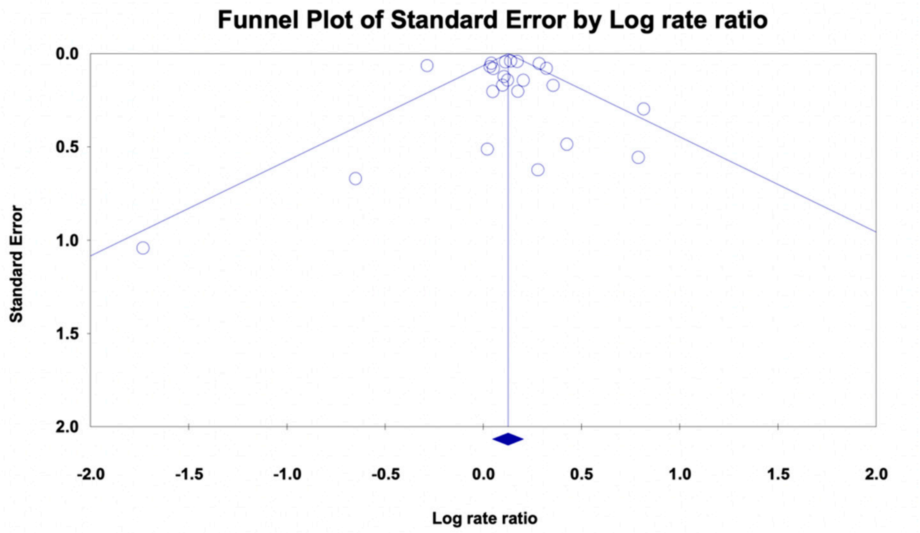

Figure S6. Sensitivity analysis for non-clinical samples reporting suicide attempt (leave-one-out method)

Suicide Attempt (non-clinical)

Study name	Point	Lower limit	Upper limit	p-Value
Brailovskaia et al., 2021	1.136	1.049	1.229	0.002
Brausch et al., 2022	1.134	1.048	1.228	0.002
G'etaz et al., 2021	1.130	1.043	1.224	0.003
Gracia et al., 2021	1.135	1.047	1.230	0.002
Kasal et al., 2022a	1.096	1.012	1.187	0.025
Kasal et al., 2022b	1.088	1.004	1.178	0.039
Kim et al., 2021	1.168	1.077	1.266	0.000
King et al., 2022	1.151	1.063	1.246	0.001
Koenig et al., 2021	1.140	1.053	1.233	0.001
Lee & Hong, 2022a	1.163	1.072	1.260	0.000
Lee & Hong, 2022b	1.157	1.068	1.255	0.000
Mayne et al., 2021	1.140	1.052	1.236	0.001
Reif - Leonhard et al., 2022	1.163	1.073	1.261	0.000
Reuter et al., 2021	1.139	1.052	1.233	0.001
S. Y. Kim et al., 2022	1.142	1.054	1.236	0.001
Sivertsen et al., 2022	1.145	1.056	1.240	0.001
Stando et al., 2022a	1.152	1.063	1.248	0.001
Stando et al., 2022b	1.144	1.053	1.243	0.001
Stando et al., 2022c	1.144	1.054	1.241	0.001
Stando et al., 2022d	1.148	1.055	1.249	0.001
Stando et al., 2022e	1.150	1.057	1.250	0.001
Stando et al., 2022f	1.151	1.060	1.251	0.001
Stando et al., 2022g	1.081	1.023	1.143	0.005
Stando et al., 2022h	1.134	1.046	1.230	0.002
Stando et al., 2022i	1.139	1.050	1.236	0.002
Stando et al., 2022j	1.136	1.047	1.233	0.002
Stando et al., 2022k	1.153	1.061	1.253	0.001
Stando et al., 2022l	1.153	1.060	1.254	0.001
Valdez-Santiago et al., 2022	1.139	1.051	1.234	0.002
Zhang et al., 2020	1.124	1.038	1.217	0.004
	1.140	1.053	1.233	0.001

Rate ratio (95% CI) with study removed

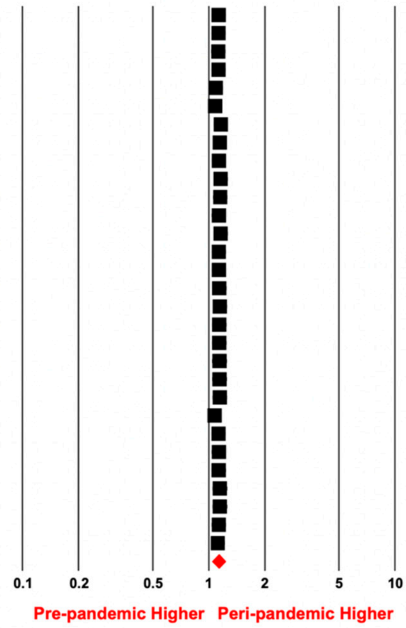

Meta Analysis

Figure S7. Sensitivity analysis for non-clinical samples reporting suicide attempt (exclude lower-quality samples)

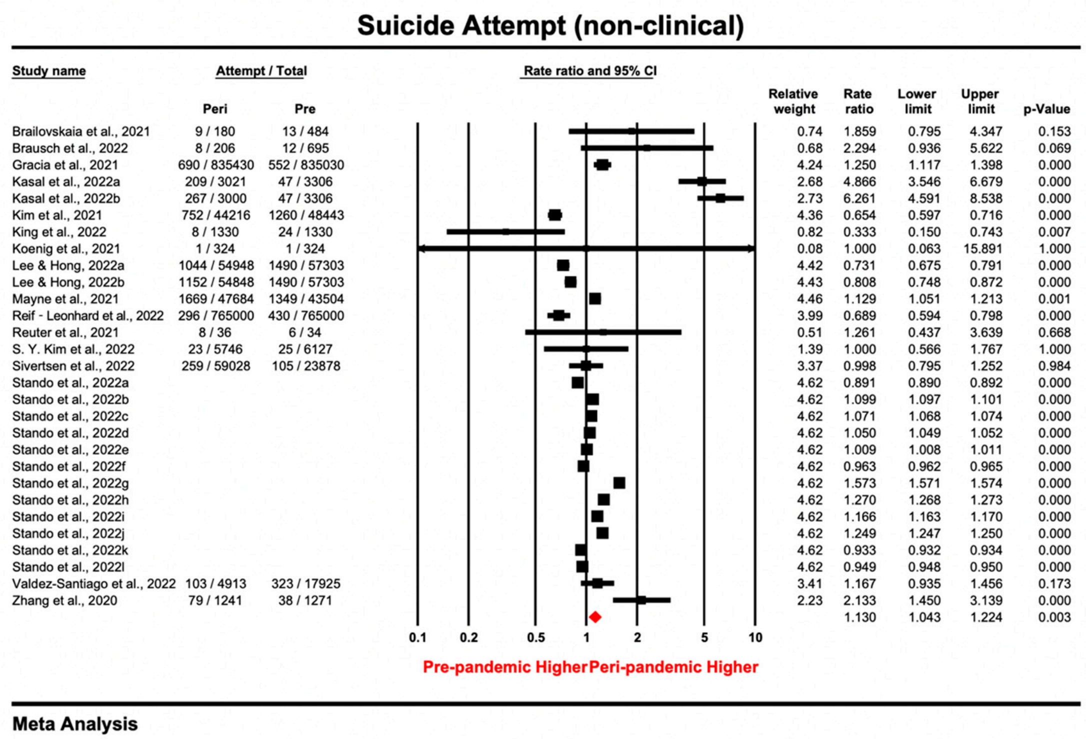

Figure S8. Funnel plot for non-clinical samples reporting suicide attempt

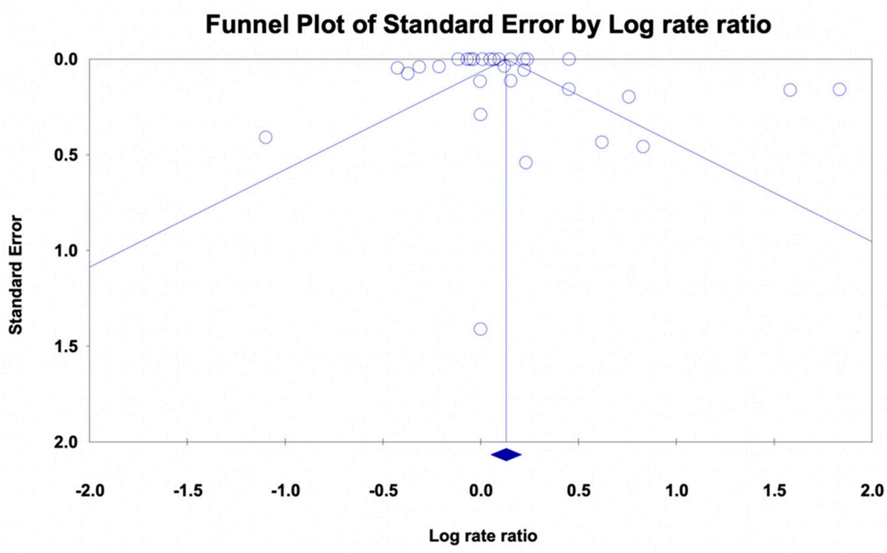

Figure S9. Sensitivity analysis for clinical samples reporting suicide attempt (leave-one-out method)

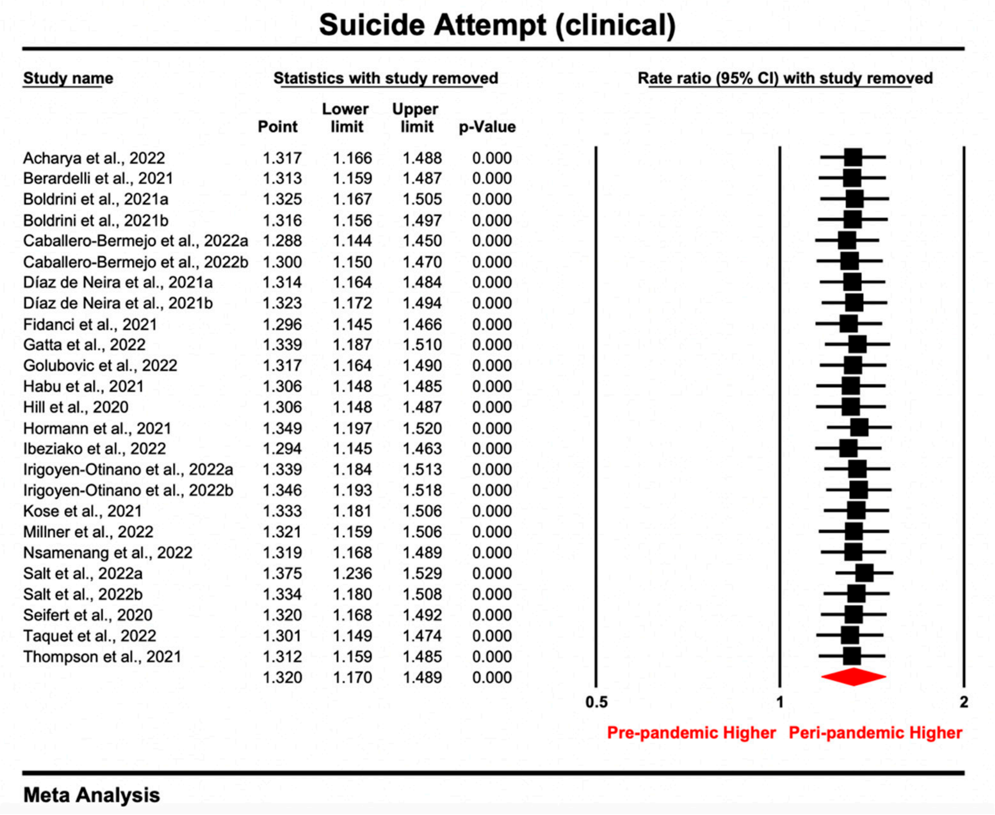

Study name	Point	Lower limit	Upper limit	p-Value
Acharya et al., 2022	1.317	1.166	1.488	0.000
Berardelli et al., 2021	1.313	1.159	1.487	0.000
Boldrini et al., 2021a	1.325	1.167	1.505	0.000
Boldrini et al., 2021b	1.316	1.156	1.497	0.000
Caballero-Bermejo et al., 2022a	1.288	1.144	1.450	0.000
Caballero-Bermejo et al., 2022b	1.300	1.150	1.470	0.000
Díaz de Neira et al., 2021a	1.314	1.164	1.484	0.000
Díaz de Neira et al., 2021b	1.323	1.172	1.494	0.000
Fidanci et al., 2021	1.296	1.145	1.466	0.000
Gatta et al., 2022	1.339	1.187	1.510	0.000
Golubovic et al., 2022	1.317	1.164	1.490	0.000
Habu et al., 2021	1.306	1.148	1.485	0.000
Hill et al., 2020	1.306	1.148	1.487	0.000
Hormann et al., 2021	1.349	1.197	1.520	0.000
Ibeziako et al., 2022	1.294	1.145	1.463	0.000
Irigoyen-Otinano et al., 2022a	1.339	1.184	1.513	0.000
Irigoyen-Otinano et al., 2022b	1.346	1.193	1.518	0.000
Kose et al., 2021	1.333	1.181	1.506	0.000
Millner et al., 2022	1.321	1.159	1.506	0.000
Nsamenang et al., 2022	1.319	1.168	1.489	0.000
Salt et al., 2022a	1.375	1.236	1.529	0.000
Salt et al., 2022b	1.334	1.180	1.508	0.000
Seifert et al., 2020	1.320	1.168	1.492	0.000
Taquet et al., 2022	1.301	1.149	1.474	0.000
Thompson et al., 2021	1.312	1.159	1.485	0.000
	1.320	1.170	1.489	0.000

Figure S10. Sensitivity analysis for clinical samples reporting suicide attempt (exclude lower-quality samples)

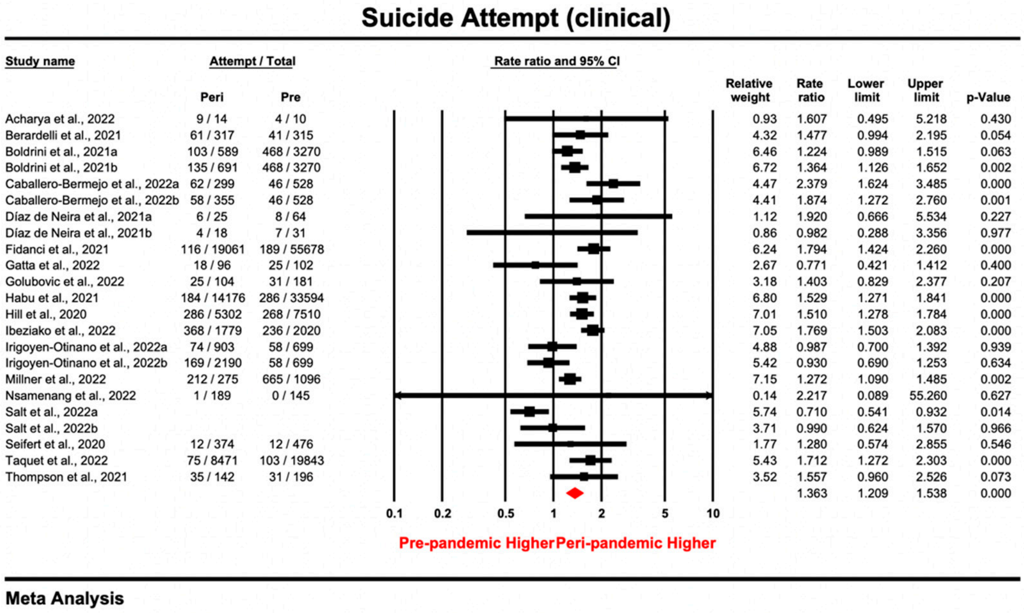

Figure S11. Funnel plot for clinical samples reporting suicide attempt

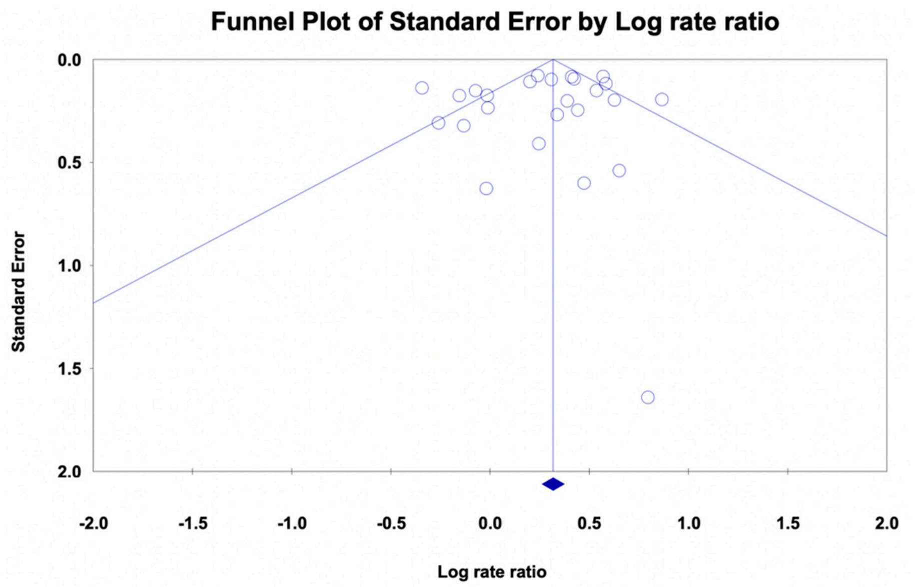

Figure S12. Sensitivity analysis for samples reporting suicide death (leave-one-out method)

Death by Suicide

Study name	Statistics with study removed			
	Point	Lower limit	Upper limit	p-Value
Acharya et al., 2022	0.913	0.831	1.003	0.059
Appleby et al., 2021	0.920	0.837	1.012	0.086
Arya et al., 2022	0.916	0.833	1.008	0.071
Chen et al., 2022	0.923	0.839	1.015	0.097
de la Torre-Luque et al., 2022	0.917	0.834	1.010	0.078
Faust et al., 2021	0.926	0.843	1.018	0.112
Gerstner et al., 2022	0.918	0.834	1.011	0.082
Larson et al., 2022a	0.931	0.847	1.024	0.141
Larson et al., 2022b	0.924	0.840	1.015	0.100
McIntyre et al., 2021	0.936	0.852	1.027	0.162
Mitchel & Li, 2021	0.926	0.840	1.020	0.120
Page et al., 2022a	0.923	0.837	1.019	0.111
Page et al., 2022b	0.924	0.837	1.019	0.113
Palacio-Mejía et al.2022a	0.920	0.836	1.013	0.089
Palacio-Mejía et al.2022b	0.919	0.835	1.012	0.086
Radeloff et al., 2021a	0.935	0.853	1.026	0.156
Radeloff et al., 2021b	0.955	0.898	1.016	0.142
Reif - Leonhard et al., 2022	0.921	0.838	1.012	0.087
Rogalska & Syrkiewicz-Switala, 2022	0.918	0.833	1.010	0.080
Rück et al., 2020	0.921	0.836	1.014	0.094
Ryu et al., 2022	0.922	0.833	1.020	0.116
Stene-Larsen et al., 2022	0.921	0.835	1.016	0.100
Tanaka & Okamoto, 2021a	0.924	0.838	1.019	0.114
Tanaka & Okamoto, 2021b	0.915	0.832	1.006	0.066
Wei et al., 2021	0.917	0.834	1.008	0.072
	0.923	0.842	1.013	0.092

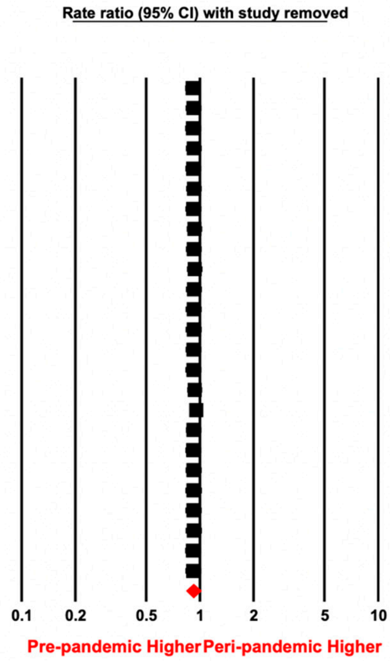

Meta Analysis

Figure S13. Sensitivity analysis for samples reporting suicide death (exclude lower-quality samples)

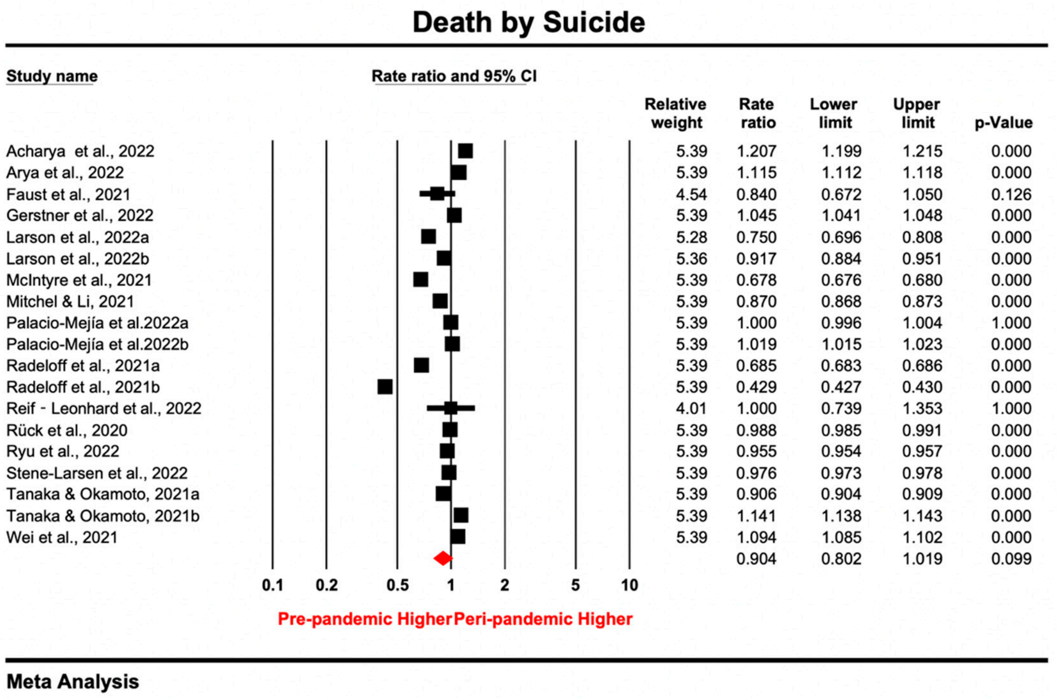

Figure S14. Funnel plot for samples reporting suicide death

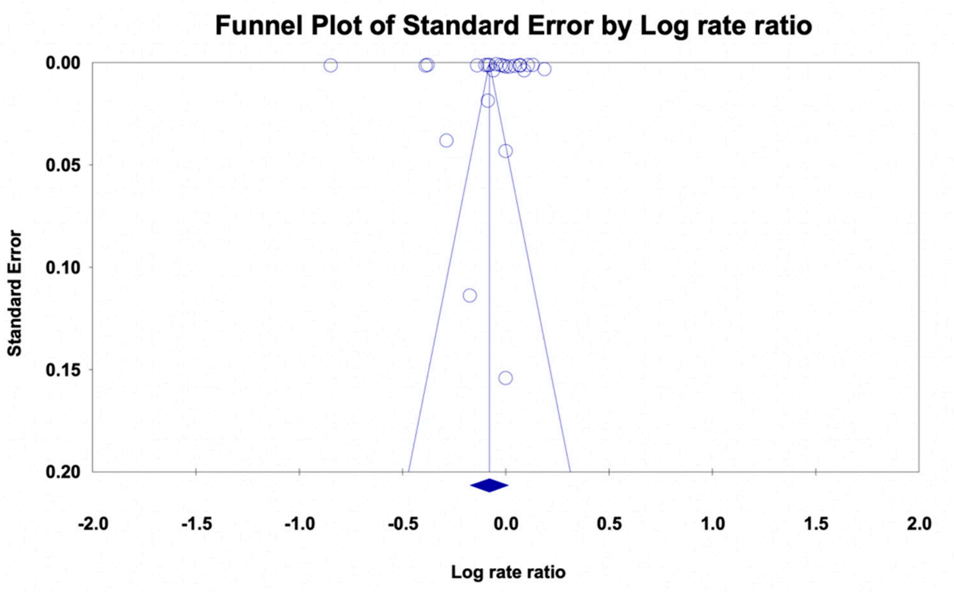

Table S1. PRISMA checklist

Section and Topic	Item #	Checklist item	Location where item is reported
**TITLE**			
Title	1	Identify the report as a systematic review.	P1
**ABSTRACT**			
Abstract	2	See the PRISMA 2020 for Abstracts checklist.	P1 (Main elements such as objectives, inclusion criteria, information sources, risk of bias assessment tool, effect size, number of included studies, summary estimates, and implications)
**INTRODUCTION**			
Rationale	3	Describe the rationale for the review in the context of existing knowledge.	P1-2
Objectives	4	Provide an explicit statement of the objective(s) or question(s) the review addresses.	P2
**METHODS**			
Eligibility criteria	5	Specify the inclusion and exclusion criteria for the review and how studies were grouped for the syntheses.	P3 (inclusion and exclusion criteria were set to screen the study design, outcomes, measurement of outcomes, publication status, and language of the potential studies)
Information sources	6	Specify all databases, registers, websites, organisations, reference lists and other sources searched or consulted to identify studies. Specify the date when each source was last searched or consulted.	P3
Search strategy	7	Present the full search strategies for all databases, registers and websites, including any filters and limits used.	P3 & File S1 (Searched terms and the combination had been presented; an example can be found in supplementary file)
Selection process	8	Specify the methods used to decide whether a study met the inclusion criteria of the	P3 (The method and procedure for selection, as well as the

Section and Topic	Item #	Checklist item	Location where item is reported
		review, including how many reviewers screened each record and each report retrieved, whether they worked independently, and if applicable, details of automation tools used in the process.	details for automation tools had been presented)
Data collection process	9	Specify the methods used to collect data from reports, including how many reviewers collected data from each report, whether they worked independently, any processes for obtaining or confirming data from study investigators, and if applicable, details of automation tools used in the process.	P3-4 (The data extraction section provided details on how data for included studies be coded)
Data items	10a	List and define all outcomes for which data were sought. Specify whether all results that were compatible with each outcome domain in each study were sought (e.g. for all measures, time points, analyses), and if not, the methods used to decide which results to collect.	P3-4 (Main outcomes, measurement tool, and collection time, etc., had been listed in data extraction section with definitions in the introduction part)
	10b	List and define all other variables for which data were sought (e.g. participant and intervention characteristics, funding sources). Describe any assumptions made about any missing or unclear information.	P4-5 (The other important variables were categorized as identification of the study, methodological, sample characteristic, and potential moderators, and were listed)
Study risk of bias assessment	11	Specify the methods used to assess risk of bias in the included studies, including details of the tool(s) used, how many reviewers assessed each study and whether they worked independently, and if applicable, details of automation tools used in the process.	P4 (An appropriate tool to assess studies reporting prevalence outcomes had been chosen, and details for the tools had been listed)
Effect measures	12	Specify for each outcome the effect measure(s) (e.g. risk ratio, mean difference) used in the synthesis or presentation of results.	P4 (An appropriate effect size had been selected to answer our question, and the calculation was specified)
Synthesis methods	13a	Describe the processes used to decide which studies were eligible for each synthesis (e.g. tabulating the study intervention characteristics and comparing against the planned groups for each synthesis (item #5)).	P4

Section and Topic	Item #	Checklist item	Location where item is reported
	13b	Describe any methods required to prepare the data for presentation or synthesis, such as handling of missing summary statistics, or data conversions.	P4
	13c	Describe any methods used to tabulate or visually display results of individual studies and syntheses.	P4
	13d	Describe any methods used to synthesize results and provide a rationale for the choice(s). If meta-analysis was performed, describe the model(s), method(s) to identify the presence and extent of statistical heterogeneity, and software package(s) used.	P4 (The model and software used to synthesize the data, and the criteria to assess heterogeneity was specified)
	13e	Describe any methods used to explore possible causes of heterogeneity among study results (e.g. subgroup analysis, meta-regression).	P4-5(The analysis conducting to explore potential heterogeneity and the way to categorise variables were specified.)
	13f	Describe any sensitivity analyses conducted to assess robustness of the synthesized results.	P4 (Two sensitivity tests were illustrated to assess the robustness of the pooled estimates)
Reporting bias assessment	14	Describe any methods used to assess risk of bias due to missing results in a synthesis (arising from reporting biases).	P4 (The information had been assessed in the risk of bias assessment)
Certainty assessment	15	Describe any methods used to assess certainty (or confidence) in the body of evidence for an outcome.	P4 (The information had been assessed in the risk of bias assessment)
**RESULTS**			
Study selection	16a	Describe the results of the search and selection process, from the number of records identified in the search to the number of studies included in the review, ideally using a flow diagram.	P5 (PRISMA flow chart had been displayed to show the process)
	16b	Cite studies that might appear to meet the inclusion criteria, but which were excluded,	Fig 1 (Detailed reasons for excluding studies in each stage

Section and Topic	Item #	Checklist item	Location where item is reported
		and explain why they were excluded.	had been specified)
Study characteristics	17	Cite each included study and present its characteristics.	P5 & Table 1-2 (Detailed information and characteristics for several aspects had been demonstrated)
Risk of bias in studies	18	Present assessments of risk of bias for each included study.	Table S2 (Score for each item of each study had been given, and summary of the results were in main text)
Results of individual studies	19	For all outcomes, present, for each study: (a) summary statistics for each group (where appropriate) and (b) an effect estimate and its precision (e.g. confidence/credible interval), ideally using structured tables or plots.	P18, 22 & 25 (The summary for three main outcomes had been organised by tables on each page)
Results of syntheses	20a	For each synthesis, briefly summarise the characteristics and risk of bias among contributing studies.	P18, 22 & 25 (Average on risk of bias score for included studies for each outcome was also given)
	20b	Present results of all statistical syntheses conducted. If meta-analysis was done, present for each the summary estimate and its precision (e.g. confidence/credible interval) and measures of statistical heterogeneity. If comparing groups, describe the direction of the effect.	P18, 22 & 25 (Overall estimates, 95% CI, p-value, and sample size for each estimate had been given)
	20c	Present results of all investigations of possible causes of heterogeneity among study results.	P19-25 (results for subgroup analysis and meta-regression had been presented with text and tables)
	20d	Present results of all sensitivity analyses conducted to assess the robustness of the synthesized results.	Figure S1, 3, 4, 6, 7, 9, 10, 12, and 13 (Each figure displays the results of two sensitivity analysis for each outcome)
Reporting biases	21	Present assessments of risk of bias due to missing results (arising from reporting biases) for each synthesis assessed.	Table S2 (The fifth item assess basically the reporting bias for included studies)
Certainty of	22	Present assessments of certainty (or confidence) in the body of evidence for each	P19-25 (See the 95% CI for each outcome and the sensitivity

Section and Topic	Item #	Checklist item	Location where item is reported
evidence		outcome assessed.	analysis results)
**DISCUSSION**			
Discussion	23a	Provide a general interpretation of the results in the context of other evidence.	P25-28 (Results were interpreted using existing evidence and theory)
	23b	Discuss any limitations of the evidence included in the review.	P27-28
	23c	Discuss any limitations of the review processes used.	P27-28 (Limitation for data extraction and subgroup analysis were discussed)
	23d	Discuss implications of the results for practice, policy, and future research.	P27-28
**OTHER INFORMATION**			
Registration and protocol	24a	Provide registration information for the review, including register name and registration number, or state that the review was not registered.	P3 (Number for PROSPERO registration was provided)
	24b	Indicate where the review protocol can be accessed, or state that a protocol was not prepared.	P3
	24c	Describe and explain any amendments to information provided at registration or in the protocol.	P3
Support	25	Describe sources of financial or non-financial support for the review, and the role of the funders or sponsors in the review.	--
Competing interests	26	Declare any competing interests of review authors.	--
Availability of data, code and	27	Report which of the following are publicly available and where they can be found: template data collection forms; data extracted from included studies; data used for all	--

Section and Topic	Item #	Checklist item	Location where item is reported
other materials		analyses; analytic code; any other materials used in the review.	

Table S2. Risk of bias assessment

Study/item	1	2	3*	4	5	6	7	8	9	Total
**Studies for suicidal ideation and attempt**										
Acharya et al. (2022)	yes	yes	yes	yes	yes	no	no	yes	yes	7
An et al. (2022)	no	yes	yes	yes	yes	yes	yes	yes	yes	8
Ayuso-Mateos et al. (2021)	yes	yes	yes	yes	yes	no	no	yes	yes	7
Berardelli et al. (2021)	yes	no	yes	yes	yes	yes	yes	yes	no	7
Berger et al. (2022)	no	yes	yes	yes	yes	no	no	yes	yes	6
Boldrini et al. (2021)	yes	no	yes	yes	yes	yes	yes	yes	yes	8
Bountress et al. (2022)	no	no	yes	yes	yes	yes	yes	yes	yes	7
Brailovskaia et al. (2021)	no	yes	yes	yes	no	yes	yes	yes	no	6
Brausch et al. (2022)	no	no	yes	yes	yes	yes	yes	yes	yes	7
Caballero-Bermejo et al. (2022)	yes	yes	yes	yes	yes	no	no	yes	yes	7
Chadi et al. (2021)	no	yes	yes	no	yes	yes	no	yes	yes	6
Danielsen et al. (2022)	yes	no	yes	yes	yes	no	no	yes	yes	6
Dı́az de Neira et al. (2021)	yes	yes	no	yes	yes	no	no	yes	yes	6
Ettman et al. (2020)	yes	no	yes	yes	yes	yes	yes	yes	no	7
Fidancı et al. (2021)	no	yes	yes	yes	yes	no	no	yes	yes	6
Gatta et al. (2022)	yes	no	yes	yes	no	yes	yes	yes	no	6
G’etaz et al. (2021)	yes	no	yes	no	yes	no	no	yes	no	4
Golubovic et al. (2022)	no	no	yes	yes	yes	yes	yes	yes	yes	7
Gracia et al. (2021)	no	yes	yes	no	yes	yes	yes	yes	yes	7
Gratz et al. (2021)	no	yes	yes	yes	yes	no	no	yes	yes	6
H. Kim et al. (2022)	yes	yes	yes	yes	yes	yes	yes	yes	yes	9
Habu et al. (2021)	yes	yes	yes	yes	yes	no	no	yes	yes	7
Hill et al. (2020)71	yes	yes	yes	yes	yes	yes	yes	yes	no	8
Horita et al. (2022)	no	yes	yes	yes	yes	no	no	yes	yes	6
Ho̎rmann et al. (2021)	yes	no	yes	yes	yes	no	no	yes	no	5
Ibeziako et al. (2022)	no	yes	yes	yes	yes	yes	no	yes	yes	7
Irigoyen-Otin̄ano et al. (2022)	yes	yes	yes	yes	yes	yes	no	yes	yes	8
Kasal et al., (2022)	yes	yes	yes	yes	yes	yes	yes	yes	yes	9
Kim et al. (2021)	yes	yes	yes	yes	yes	no	no	yes	yes	7
King et al. (2022)	yes	yes	yes	yes	yes	yes	yes	yes	yes	9
Knudsen et al. (2021)	yes	yes	yes	yes	yes	yes	yes	yes	yes	9
Koenig et al. (2021)	yes	yes	yes	yes	yes	yes	yes	yes	yes	9
Kose et al. (2021)	no	yes	yes	no	yes	no	no	yes	yes	5
Lee & Hong (2022)	yes	yes	yes	yes	yes	yes	yes	yes	yes	9
Liu et al. (2022)	yes	yes	yes	yes	yes	yes	yes	yes	yes	9
Mayne et al. (2021)	no	no	yes	yes	yes	yes	yes	yes	no	6
McLoughlin et al. (2022)	no	yes	no	yes	yes	no	no	yes	yes	5
Millner et al. (2022)	no	no	yes	yes	yes	yes	no	yes	yes	6
Nichter et al. (2021)	yes	yes	yes	yes	yes	yes	yes	yes	yes	9
Nsamenang et al. (2022)	yes	yes	yes	yes	yes	no	no	yes	yes	7

Reif-Leonhard et al. (2022)	yes	yes	yes	no	yes	yes	yes	yes	yes	8
Reuter et al. (2021)	no	yes	yes	yes	yes	no	no	yes	yes	6
S. Y. Kim et al. (2022)	yes	yes	yes	yes	yes	yes	yes	yes	yes	9
Sacco et al. (2022)	no	yes	yes	yes	yes	yes	yes	yes	yes	8
Salt et al. (2022)	yes	yes	yes	yes	yes	yes	no	yes	yes	8
Seifert et al. (2020)	no	no	yes	yes	yes	yes	yes	yes	no	6
Sivertsen et al. (2022)	yes	yes	yes	yes	yes	yes	yes	yes	no	8
Stan´do et al. (2022)	yes	yes	yes	yes	yes	yes	yes	yes	yes	9
Taquet et al. (2022)	yes	no	yes	yes	yes	yes	yes	yes	no	7
Thompson et al. (2021)	no	no	yes	yes	yes	yes	yes	yes	no	6
Valdez-Santiago et al. (2022)	yes	yes	yes	yes	yes	yes	yes	yes	no	8
Zhang et al. (2020)	no	no	yes	yes	yes	yes	no	yes	yes	6
Zhu et al. (2021)	no	no	yes	yes	yes	yes	yes	yes	no	6
**Studies for death by suicide**										
Acharya et al. (2022)	yes	yes	yes	yes	yes	yes	yes	yes	yes	9
Appleby et al. (2021)	no	yes	yes	no	yes	no	no	yes	yes	5
Arya et al. (2022)	yes	yes	yes	yes	yes	no	no	yes	yes	7
Chen et al. (2022)	yes	yes	yes	no	yes	no	no	yes	yes	6
de la Torre-Luque et al. (2022)	yes	yes	yes	no	yes	no	no	yes	yes	6
Faust et al. (2021)	yes	yes	yes	yes	yes	no	no	yes	yes	7
Gerstner et al. (2022)	yes	yes	yes	yes	yes	yes	yes	yes	yes	9
Larson et al. (2022)	yes	yes	yes	yes	yes	yes	yes	yes	yes	9
McIntyre et al. (2021)	yes	yes	yes	no	yes	yes	yes	yes	yes	8
Mitchel & Li (2021)	yes	yes	yes	yes	yes	no	no	yes	yes	7
Page et al. (2022)	yes	yes	yes	yes	yes	no	no	no	yes	6
Palacio-Mejía et al. (2022)	yes	yes	yes	yes	yes	yes	yes	yes	yes	9
Radeloff et al. (2021)	yes	yes	yes	no	yes	yes	yes	yes	yes	8
Reif-Leonhard et al. (2022)	no	yes	yes	yes	yes	yes	yes	yes	yes	8
Rogalska& Syrkiewicz-Switała (2022)	yes	yes	yes	no	yes	no	no	yes	yes	6
Rück et al. (2020)	yes	yes	yes	no	yes	yes	yes	yes	yes	8
Ryu et al. (2022)	yes	yes	yes	yes	yes	yes	yes	yes	yes	9
Stene-Larsen et al. (2022)	yes	yes	yes	yes	yes	yes	yes	yes	yes	9
Tanaka & Okamoto (2021)	yes	yes	yes	yes	yes	yes	yes	yes	yes	9
Wei et al. (2021)	yes	yes	yes	yes	yes	yes	yes	yes	yes	9

* The third item assessed the bias on sample size. All data for death by suicide were national or regional level, which would be of low risk of bias. For studies reporting suicidal ideation and attempt, if sample size calculation was conducted, yes will be marked for this item; if no prior estimation, we conducted an estimation based on the formula $N = Z^2*p*(1-p)/d^2$ according to JBI Critical Appraisal Instrument, to see if the sample size of the included studies fulfilled the responding criterion. The prevalence of suicidal ideation and suicide attempt ranged from 2% to 50% and 0.3% to 64% in our included samples, thus, the minimum sample size for ideation and attempt would be around 30 and 5.

Table S3. Summary of meta-regression for non-clinical and clinical samples reporting suicidal ideation

Covariate	Beta	Standard Error	95% Lower	95% Upper	*p* - value
Female proportion	-0.17	0.41	-0.98	0.65	0.69
Study quality	0.06	0.36	-1.03	0.39	0.38
Female proportion	0.40	0.45	-0.48	1.27	0.37
Study quality	0.08	0.04	0.01	0.16	0.03

Table S4. Summary of meta-regression for non-clinical and clinical samples reporting suicide attempt

Covariate	Beta	Standard Error	95% Lower	95% Upper	*p* - value
Female proportion	0.13	0.11	-0.08	0345	0.21
Study quality	-0.03	0.30	-0.19	0.94	0.39
Female proportion	-0.50	0.45	-1.39	0.38	0.27
Study quality	-0.02	0.07	-0.15	0.11	0.79

Table S5. Summary of meta-regression for samples reporting death by suicide

**Moderators**	**Beta**	**Standard Error**	**95% Lower**	**95% Upper**	***p*-value**
GDP (Peri/Pre)	-0.31	0.66	-1.60	0.97	0.63
Unemployment rate (Peri/Pre)	-0.01	0.095	-0.20	0.17	0.91
Resilience score	-0.01	0.01	-0.02	0.01	0.38
Stringency index	0.003	0.003	-0.003	0.01	0.33
Containment and health index	-0.00009	0.004	-0.008	0.008	0.98
Economic support index	-0.0008	0.001	-0.004	0.002	0.59

File S1. Full search strategy

A. **Search terms** (Following the CoCoPop mnemonic)

- **Condition**

  (suicid* OR “suicidal ideation” OR “suicidal thoughts” OR “suicidal plan” OR “suicide attempt” OR “completed suicide” OR “death by suicide”) AND (COVID* OR coronavirus OR “2019-ncov” OR “sars-cov-2” OR “cov-19” OR “2019 pandemic”)

- **Context**

  "COVID*" OR coronavirus OR "2019-ncov" OR "sars-cov-2" OR "cov-19" OR "2019 pandemic"

B. **Search strategy** (An example from PubMed)

Search: ((suicid*[Title/Abstract] OR "suicidal ideation"[Title/Abstract] OR "suicidal thoughts"[Title/Abstract] OR "suicidal plan"[Title/Abstract] OR "suicide attempt"[Title/Abstract] OR "completed suicide"[Title/Abstract] OR "death by suicide"[Title/Abstract])) AND ((COVID*[Title/Abstract] OR coronavirus[Title/Abstract] OR "2019-ncov"[Title/Abstract] OR "sars-cov-2"[Title/Abstract] OR "cov-19"[Title/Abstract] OR "2019 pandemic"[Title/Abstract]))

("suicid*"[Title/Abstract] OR "suicidal ideation"[Title/Abstract] OR "suicidal thoughts"[Title/Abstract] OR "suicidal plan"[Title/Abstract] OR "suicide attempt"[Title/Abstract] OR "completed suicide"[Title/Abstract] OR "death by suicide"[Title/Abstract]) AND ("covid*"[Title/Abstract] OR "coronavirus"[Title/Abstract] OR "2019-ncov"[Title/Abstract] OR "sars-cov-2"[Title/Abstract] OR "cov-19"[Title/Abstract] OR "2019 pandemic"[Title/Abstract])
